# Preventing the Galvanic Replacement Reaction toward Unconventional Bimetallic Core–Shell Nanostructures

**DOI:** 10.3390/molecules28155720

**Published:** 2023-07-28

**Authors:** Kai Liu, Zhun Qiao, Chuanbo Gao

**Affiliations:** Frontier Institute of Science and Technology, Xi’an Jiaotong University, Xi’an 710054, China; liu.k.n@xjtu.edu.cn (K.L.); qiaozhun@stu.xjtu.edu.cn (Z.Q.)

**Keywords:** galvanic replacement prevention, core–shell nanostructure, noble metal, optical property, catalytic property

## Abstract

A bimetallic core–shell nanostructure is a versatile platform for achieving intriguing optical and catalytic properties. For a long time, this core–shell nanostructure has been limited to ones with noble metal cores. Otherwise, a galvanic replacement reaction easily occurs, leading to hollow nanostructures or completely disintegrated ones. In the past few years, great efforts have been devoted to preventing the galvanic replacement reaction, thus creating an unconventional class of core–shell nanostructures, each containing a less-stable-metal core and a noble metal shell. These new nanostructures have been demonstrated to show unique optical and catalytic properties. In this work, we first briefly summarize the strategies for synthesizing this type of unconventional core–shell nanostructures, such as the delicately designed thermodynamic control and kinetic control methods. Then, we discuss the effects of the core–shell nanostructure on the stabilization of the core nanocrystals and the emerging optical and catalytic properties. The use of the nanostructure for creating hollow/porous nanostructures is also discussed. At the end of this review, we discuss the remaining challenges associated with this unique core–shell nanostructure and provide our perspectives on the future development of the field.

## 1. Introduction

Bimetallic nanocrystals are receiving increasing interest in optics [1,2,3], catalysis [4,5,6,7,8,9,10], and many other fields [11,12,13,14,15]. For example, bimetallic nanocrystals are an excellent class of catalysts for many industrial processes and energy conversion thanks to their intriguing catalytic properties arising from the electronic and geometric interactions between constituent metals [8,9,10]. Among the different types of bimetallic nanocrystals, core–shell structured nanocrystals have attracted significant attention in recent years [1,15,16,17,18]. These core–shell nanostructures may show many advantages, including convenient shape and facet control [3,6,19], the high utilization efficiency of the shell metals [17,20,21], and well-controlled properties due to the core–shell interactions [9,19,22,23].

Generally, most bimetallic core–shell nanostructures investigated to date are those with noble metal cores, such as Au, Pd, and Pt [16,18,24,25]. Such metal cores are highly stable, and thus can well resist oxidative etching by the precursor of the shell metal. When less stable metals are employed as the cores, a galvanic replacement reaction readily occurs [26,27,28,29]. Upon mixing the less-stable-metal nanocrystals (cores) with noble metal salts (shell metal precursors), the less-stable-metal nanocrystals are oxidatively etched, while the noble metal salt is reduced and deposited onto the surface of the less-stable-metal nanocrystals. Eventually, the galvanic replacement reaction leads to hollow nanostructures or fully disintegrated ones [29,30,31,32,33]. As a result, it has been challenging to synthesize core–shell nanostructures with less-stable-metal cores and noble metal shells. The lack of this kind of unconventional core–shell nanostructure has restricted the pursuit of new materials and properties.

Recently, a few research groups have made significant progress in preventing the galvanic replacement reaction in pursuit of unconventional bimetallic core–shell nanostructures [21,22,34,35,36,37,38,39,40,41,42,43,44,45,46,47]. The strategies developed to date generally fall into two categories. The first category focuses on reaction thermodynamics [21,22,34,35,36,37,38,39,40]. An extra ligand is usually introduced into the synthesis system to coordinate with the noble metal salt. As a result, the reduction potential of the noble metal salt is decreased, making it less prone to react with the less-stable-metal nanocrystals. The other category of strategies focuses on the reaction kinetics [41,42,43,44,45,46,47]. A strong reducing agent is usually introduced into the reaction system so that the reduction of the noble metal salt by the reducing agent becomes more predominant compared to the galvanic replacement reaction. Either way, the resulting core–shell nanocrystals with less-stable-metal cores and noble metal shells showed unique optical and catalytic properties, highlighting great potential impacts on the respective fields.

Herein, we provide a brief summary of recent progress in preventing the galvanic replacement reaction to afford unconventional bimetallic core–shell nanostructures, including results from a few research teams and those from our group. The effects of the core–shell nanostructure on stabilizing the core metal nanocrystals [12,48], creating unique optical and catalytic properties [4,19,21,46,49], and building various hollow nanostructures or frameworks are also discussed [36,37,50,51,52]. Finally, we provide a personal perspective on future research on the synthesis and applications of these novel bimetallic core–shell nanostructures. We expect such a discussion to give rise to new thoughts and ideas in advancing the synthetic chemistry of noble metal nanostructures with peculiar properties and wide applications.

## 2. Galvanic Replacement and Its Prevention to Synthesize Unconventional Core–Shell Nanostructures: The Case of Ag@M (M: Au, Pt, Pd, etc.) Nanostructures

### 2.1. Galvanic Replacement Reaction

The galvanic replacement reaction is an electrochemical process that involves the oxidation of one metal by the ions of another metal with a higher reduction potential [2,27,33]. Table 1 lists the standard reduction potentials (*E*^0^) of representative metal salts [53]. The driving force for a galvanic replacement reaction is the reduction potential difference between two metals, with one metal acting principally as the cathode and the other metal as the anode. Thus, the process of the galvanic replacement reaction can be divided into two half-reactions: At the anode, metal nanocrystals (*M*_1_) lose electrons and become oxidized:

*M*_1_ → *M*_1_*^m^*^+^ + *m*e^−^

At the cathode, metal ions (M_2_^n+^) gain electrons and become reduced, followed by deposition on the surface of the *M*_1_ nanocrystals:

*M*_2_^n+^ + *n*e^−^ → *M*_2_

**Figure 1:**
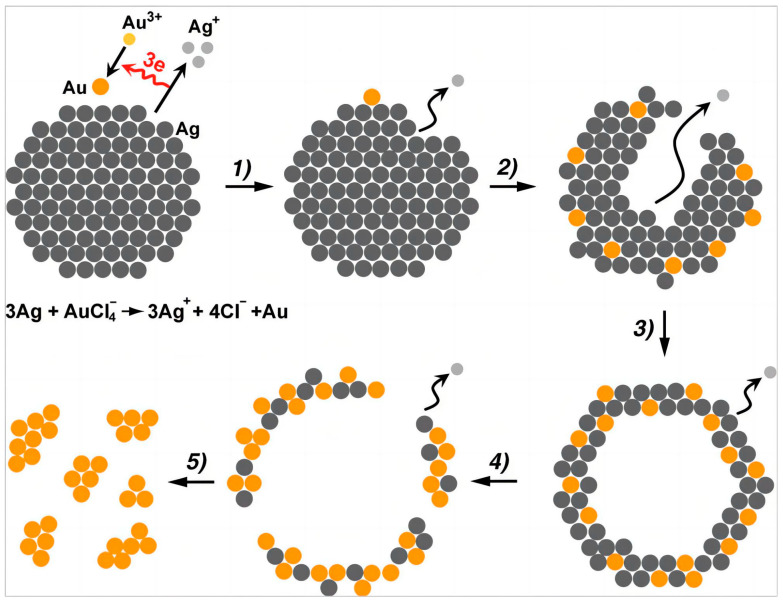
A scheme of the galvanic replacement reaction between Ag nanocrystals and Au salt (HAuCl_4_) in an aqueous solution, illustrating the morphological and structural changes at different reaction stages. (1) One Au atom is deposited onto the nanocrystal, accompanied by the removal of three Ag atoms. (2,3) Continuous deposition of Au and removal of Ag lead to an Au–Ag alloy hollow nanostructure. (4,5) The hollow nanostructure is further disintegrated by extensive galvanic replacement reaction. Reproduced from ref. [33]. Copyright (2013), with permission from Wiley-VCH.

In the case of Ag nanocrystals, they readily react with noble metal salts, such as HAuCl_4_, via a galvanic replacement reaction (Figure 1) [33]. This is because the standard reduction potential of AuCl_4_^−^ + 3e^−^ = Au + 4Cl^−^ (*E*^0^ = 1.002 V vs. SHE) is substantially larger than that of Ag^+^ (Ag^+^ + e^−^ = Ag, *E*^0^ = 0.7996 V). Thus, the oxidation of Ag nanocrystals accompanying the reduction of AuCl_4_^−^ becomes a thermodynamically favorable and spontaneous process. The Au atoms are then deposited onto the surface of Ag nanocrystals, which stabilizes the Ag surface. This process thus causes etching of Ag, preferentially from the interior of the nanocrystals, forming hollow nanostructures [26,27,28,29,30,32,40,54]. The galvanic replacement reaction also causes the mixing of Au and Ag to create a random alloy [29,32]. All these features make it a significant challenge to synthesize core−shell nanostructures, each containing a less-stable-metal core and a noble metal shell, with controllable arrangements of metal atoms.

Multiple efforts have been made to prevent the galvanic replacement reaction for the synthesis of unconventional core–shell nanostructures. Taking the growth of Au or platinum group metals (PGM, such as Pt and Pd) on Ag nanocrystals as an example, two strategies have been proven effective in suppressing the galvanic replacement, i.e., thermodynamic control strategies and kinetic control strategies. In the following sections, we briefly discuss the concepts behind these two strategies.

### 2.2. Thermodynamic Control Strategy

The origin of a galvanic replacement reaction is the reduction potential difference between the metals used. Therefore, it is straightforward to prevent a galvanic replacement reaction by decreasing the reduction potential of the noble metal salt to be deposited on the nanocrystals of less stable metals, such as Ag. In principle, the reduction potential of the metal salt is closely related to the coordination environment. When coordinating with a strong ligand, the concentration of free metal ions is significantly decreased, due to the coordination—dissociation equilibrium, leading to a significantly lower reduction potential. In other words, the standard reduction potential of a metal–ligand complex can be significantly lower than that of its free ion counterpart. Table 2 lists the standard reduction potentials of some typical noble metal ions coordinated to different types of ligands. More data can be found in the *CRC Handbook of Chemistry and Physics* [53]. Ligand engineering provides a convenient way to regulate the reduction potential of the noble metal salt for its galvanic-replacement-free reduction and deposition on less-stable-metal nanocrystals.

**Figure 2:**
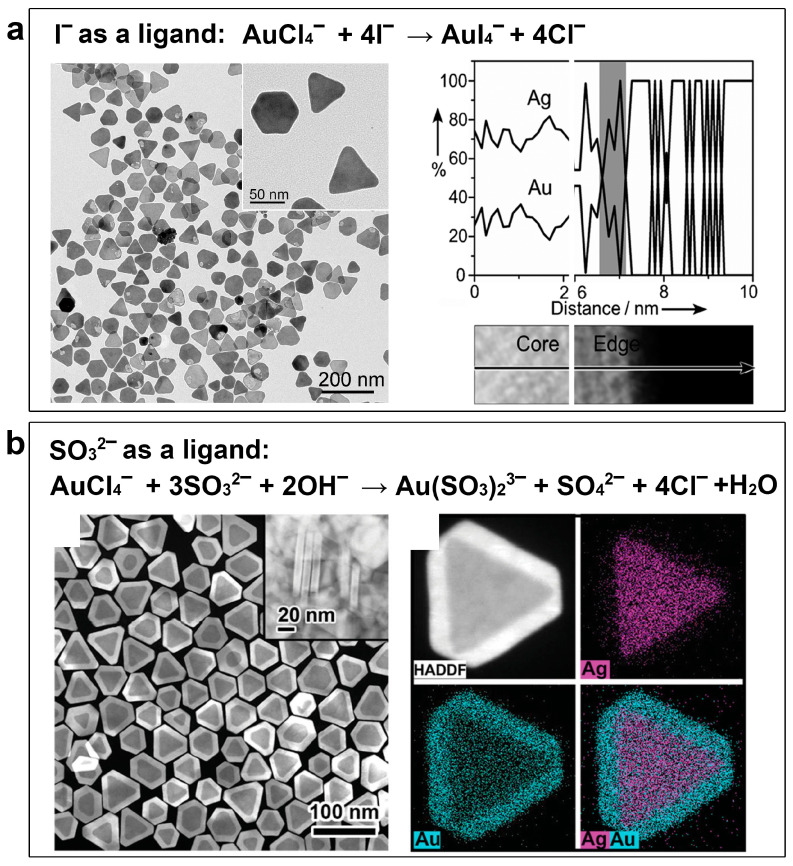
Suppressing the galvanic replacement reaction in the synthesis of Ag@Au core–shell nanoplates by coordinating the Au salt to inorganic anion-type ligands. (**a**) Ligand I^−^. TEM image and EDS elemental line distribution. Reproduced from ref. [34]. Copyright (2012), with permission from Wiley-VCH. (**b**) Ligand SO_3_^2−^. HAADS–STEM image and EDS elemental mapping. Reproduced from ref. [35]. Copyright (2015), with permission from Wiley-VCH.

Dating back to the year 2012, our research team led by Yin first demonstrated the feasibility of this thermodynamic control strategy by depositing a thin layer of Au on Ag nanoplates for stabilizing the Ag nanoplates in surface plasmon resonance (SPR) biosensing applications [34] (Figure 2a). In that work, an inorganic anion, I^−^, was introduced into the synthesis system, which served as a strong ligand for the Au salt, HAuCl_4_, to decrease its reduction potential. The standard reduction potential of the Au salt was reduced from 1.002 V (AuCl_4_^−^) to 0.56 V (AuI_4_^−^), which is even lower than that of Ag^+^ (0.80 V). Thus, the galvanic replacement reaction between Ag nanoplates and Au precursor can be prevented. In this way, Ag@Au core–shell nanoplates were successfully obtained from this synthesis. As shown in Figure 2a, the original triangular/hexagonal shapes of the Ag nanoplates were without significant hollowing. An elemental distribution analysis by energy-dispersive X-ray spectroscopy (EDS) confirmed the uniform deposition of Au on the Ag nanoplate surface. However, in this synthesis, the introduction of I^−^ may also help the etching of Ag by forming AgI, a stable coordination compound. Therefore, this synthesis failed to produce a thick Au layer on the surface of Ag nanocrystals. To make the synthesis more robust, our group further developed an improved thermodynamic control strategy by introducing sulfite (SO_3_^2−^) as a ligand [35] (Figure 2b). Sulfite can strongly coordinate with Au salt to form Au(SO_3_)_2_^3−^, which possesses an even lower standard reduction potential of 0.111 V. In addition, the ligand sulfite is mild and proven incapable of triggering the ligand-assisted oxidative etching of the Ag nanoplates. In this way, controllable epitaxial growth of Au on Ag nanocrystals was achieved layer by layer without involving a galvanic replacement reaction, leading to the formation of well-defined Ag@Au core–shell nanoplates. The high-angle annular dark field scanning transmission electron microscopy (HAADF−STEM) image and the EDS elemental mappings in Figure 2b reveal the core–shell nanostructure, confirming the absence of a galvanic replacement reaction during the synthesis.

**Figure 3:**
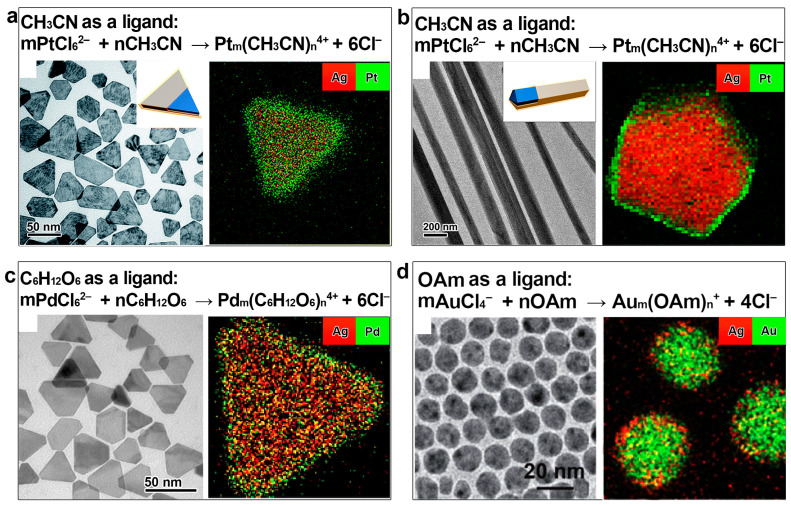
Suppressing the galvanic replacement reaction in synthesizing Ag@M (M: Pt, Pd, Au) core–shell nanocrystals by coordinating the noble metal salts to organic ligands. (**a**,**b**) Ag@Pt core–shell nanoplates and nanowires synthesized with the ligand of CH_3_CN. Reproduced from ref. [36,37]. Copyright (2018), with permission from the Royal Society of Chemistry. (**c**) Ag@Pd core–shell nanoplates synthesized with the ligand of glucose. Reproduced from ref. [22]. Copyright (2021), with permission from the Royal Society of Chemistry. (**d**) Ag@Au core–shell nanoparticles synthesized with the ligand of OAm. Reproduced from ref. [40]. Copyright (2022), with permission from the American Chemical Society.

Apart from the inorganic anion-type ligands, some organic compounds can also be employed as ligands to coordinate with noble metal salts to regulate their reduction potentials and prevent galvanic replacement reactions for the synthesis of the unconventional core–shell nanostructures. In pursuit of Ag@Pt core–shell nanocrystals, our group successfully prevented a galvanic replacement reaction between Ag nanocrystals and the Pt salt by employing acetonitrile (CH_3_CN) as the ligand [36,37]. Nuclear magnetic resonance (NMR) confirmed that the nitrile group effectively coordinates with the Pt salt. As a result, Ag@Pt core–shell nanoplates and nanowires were successfully obtained without involving a galvanic replacement reaction (Figure 3a,b). Similarly, we discovered that Pd could grow on Ag nanocrystals to form a Ag@Pd core–shell nanostructure when glucose (C_6_H_12_O_6_) was used as the ligand to the Pd salt (Figure 3c) [22]. The solution containing NaPdCl_4_ and C_6_H_12_O_6_ showed a gradual increase in ultraviolet–visible (UV–vis) absorbance, which suggests the formation of the coordination compound. In a similar way, Li found that oleylamine (OAm) and triphenylphosphine are also excellent ligands for Au, Pd, and Pt salts when targeting their galvanic-replacement-free growth on Ag nanoparticles (Figure 3d) [40]. All these facts confirm the versatility of the thermodynamic control strategy in synthesizing unconventional Ag@M core–shell nanostructures.

### 2.3. Kinetic Control Strategy

**Figure 4:**
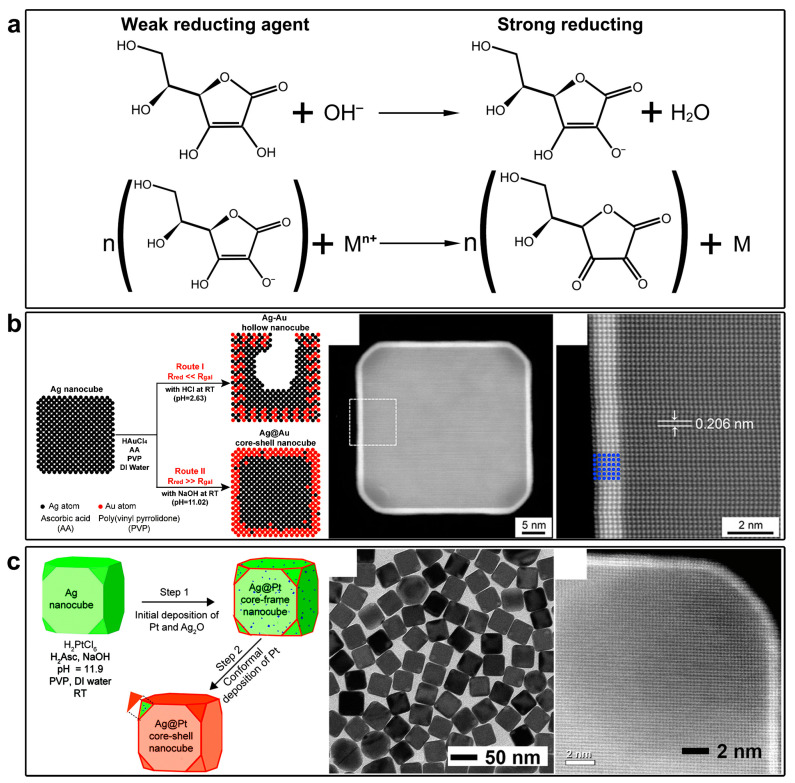
Preventing the galvanic replacement reaction by introducing a competing reduction parallel reaction. (**a**) Tuning the reducing power of AA by pH. (**b**) Preventing the galvanic replacement reaction between Ag nanocubes and HAuCl_4_ in the presence of AA at high pH values. Reproduced from ref. [41]. Copyright (2014), with permission from the American Chemical Society. (**c**) Preventing the galvanic replacement reaction between Ag nanocubes and H_2_PtCl_6_ by a similar method. Reproduced from ref. [42]. Copyright (2017), with permission from the Royal Society of Chemistry.

In 2014, Qin and co-workers reported a kinetic control strategy in the synthesis of Ag@Au core–shell nanocubes without involving a significant galvanic replacement reaction (Figure 4) [41]. Success in preventing the galvanic replacement reaction relies on the introduction of a strong reducing agent. They found that by increasing pH, ascorbic acid (AA) as a reducing agent will be deprotonated, which shows significantly enhanced reducing power (Figure 4a). In this case, the Au salt (HAuCl_4_) will react preferentially with the deprotonated AA rather than the Ag nanocrystals. The galvanic replacement reaction becomes less competitive and thus significantly suppressed. Using this kinetic control strategy, the authors demonstrated the synthesis of an ultrathin Au shell of 0.6 nm thickness on the surface of Ag nanocubes, forming a well-defined core–shell nanostructure (Figure 4b). They demonstrated that the same principle could be extended to grow conformal, ultrathin shells of Pt on the surface of Ag nanocubes for the generation of Ag@Pt core–shell nanocubes with a shell as thin as three atomic layers (Figure 4c) [42]. Thus, introducing a faster reduction parallel reaction to compete with the galvanic replacement reaction is another effective and general method to suppress the galvanic replacement reaction in pursuit of unconventional Ag@M core–shell nanostructures.

**Figure 5:**
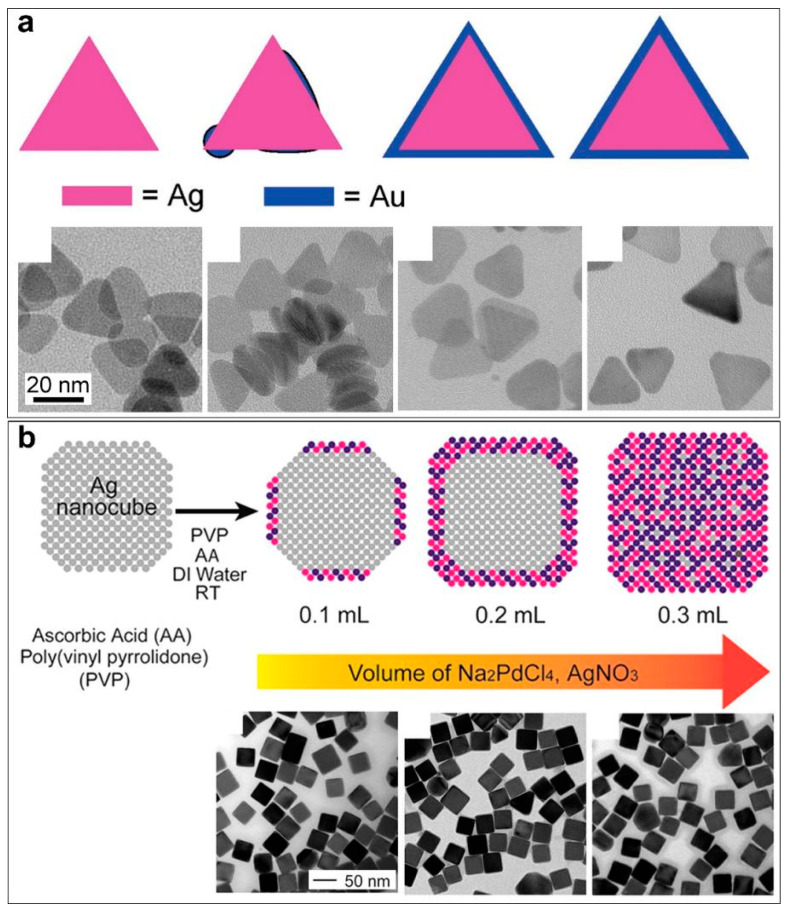
Preventing the galvanic replacement reaction by lowering metal salt concentrations. (**a**) Synthesis of Ag@Au nanoprisms by slowly dropping Au salt in Ag nanoprisms. Reproduced from ref. [55]. Copyright (2009), with permission from the American Chemical Society. (**b**) Synthesis of Ag@Pd-Ag nanocubes by slowly adding low concentrations of Pd and Ag salts. Reproduced from ref. [51]. Copyright (2015), with permission from the American Chemical Society.

Apart from increasing the power of the reducing agent, similar kinetic control can be achieved by lowering the concentration of the metal salt (Figure 5). This leads to an increase in the reducing agent/metal salt ratio, thus making the parallel reduction of the metal salt by the reducing agent more competitive than the galvanic replacement reaction. Gun’ko and co-workers reported an epitaxial deposition of a thin layer of Au on Ag nanoprisms by dropwise addition of low concentrations of HAuCl_4_ (0.5 mM) in Ag nanoprisms and ascorbic acid solution (Figure 5a) [55]. Qin and co-workers successfully prevented the galvanic replacement reaction between Ag nanocubes and Na_2_PdCl_4_ in the synthesis of Ag@Pd-Ag core–shell nanocubes by adding Na_2_PdCl_4_ (0.2 mM) and AgNO_3_ (0.1 mM) into an aqueous suspension of Ag nanocubes in the presence of AA at a slow injection rate (Figure 5b) [51]. By increasing the total volume of precursor solutions added into the reaction system, the growth pattern can be controlled so that Pd and Ag atoms are progressively deposited on the edges, corners, and then side faces of the Ag nanocubes.

## 3. Synthesis of 3d Transition Metal@noble Metal Core–Shell Nanostructures without Involving a Galvanic Replacement Reaction

Compared with the Ag cores discussed above, 3d transition metals, such as Fe, Co, Ni, and Cu, have lower prices and even stronger electronic interactions with noble metals. These 3d transition metals may induce strong compressive strains in the coherent noble metal shells, thus broadening their d-band, downshifting the d-band center position, and imposing a significant impact on their catalytic properties [8,49,56]. The overall core–shell nanomaterials show low cost and high stability, and thus may find even broader applications. However, it is a great challenge to prevent the galvanic replacement reaction between the 3d transition metals and the noble metal salt due to the former’s ultralow reduction potential values (e.g., Cu^2+^ + 2 e^−^ = Cu, *E*^0^ = 0.340 V; Ni^2+^ + 2 e^−^ = Ni, *E*^0^ = −0.257 V) and thus the huge reduction potential gap from the noble metal salts [53]. Basically, both the thermodynamic and the kinetic control strategies may be applicable to prevent the galvanic replacement reaction. Between the two, the thermodynamic control strategy, i.e., the reduction potential engineering of the noble metal salt, should be more reliable, considering the huge reduction potential gaps. In addition, the 3d transition metals are prone to oxidization, making it difficult to achieve a controlled synthesis. Therefore, during the synthesis, great care should also be taken in handling the 3d transition metal nanocrystals to avoid their possible oxidation by O_2_ dissolved in the solution or in ambient air.

**Figure 6:**
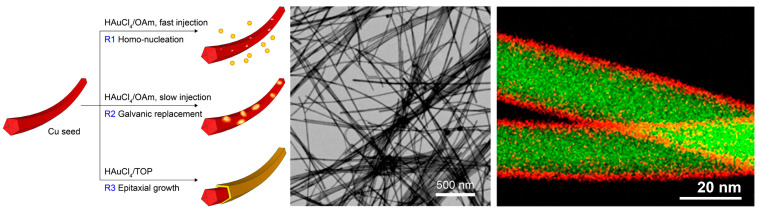
Preventing the galvanic replacement reaction between Cu nanowires and Au salts with the ligand of TOP. Scheme of the reaction mechanism, TEM image, and EDS elemental mapping of the resulting Cu@Au core–shell nanowires. Reproduced from ref. [38]. Copyright (2017), with permission from the American Chemical Society.

Yang et al. found that the galvanic replacement reaction between Cu nanowires and Au salts can be prevented by employing trioctylphosphine (TOP) as a ligand for the Au salt (Figure 6) [38]. They found that TOP can modify the reaction thermodynamics by reducing the reduction potential of Au^3+^ and thus changing the reduction kinetics of the Au precursor. With appropriate reaction kinetics, Cu@Au core–shell nanowires were successfully prepared. The TEM image and EDS elemental mapping well confirmed the unconventional core–shell nanostructure.

**Figure 7:**
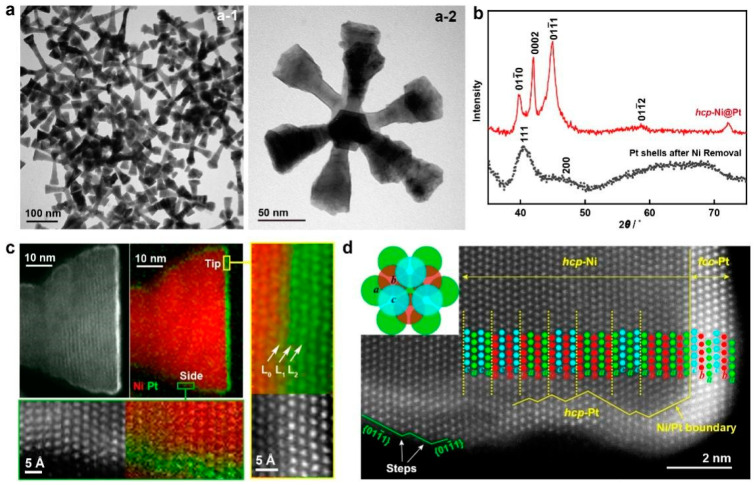
Preventing the galvanic replacement reaction between *hcp*−Ni nanobranches and the Pt salts with the aid of the ligand of OAm. (**a**) TEM images. (**a-1**) A low-magnification TEM image. (**a-2**) TEM image of a branched Ni nanocrystal. (**b**) XRD patterns of the *hcp*−Ni@Pt core–shell nanobranches and Pt shells after etching of Ni. (**c**,**d**) *C_s_*-corrected HR-STEM analysis. (**c**) Atomic-resolution EELS mappings acquired at the tip and side of the nanobranches. (**d**) Phase analysis of the nanobranches. Inset: Scheme of the atomic arrangement with *a*, *b*, *c* indicating different layers of atoms. Reproduced from ref. [21]. Copyright (2023), with permission from Springer Nature.

In addition to Cu, Ni is also a good candidate for the 3d transition metal template, possessing both face-centered cubic (*fcc*) and hexagonal close-packed (*hcp*) crystal phases [57,58]. To address the challenge of galvanic replacement with noble metal salts, our group developed a competent strategy by introducing oleylamine (OAm) as a strong ligand to the Pt salt [21]. Moreover, the synthesis was carried out at a high temperature (144 °C) with a low injection rate of Pt precursor, thus introducing extra kinetic control into the synthesis. By this means, the coherent growth of a Pt skin on *hcp*−Ni nanobranches was successfully achieved without involving a galvanic replacement reaction (Figure 7). The core–shell structure can be confirmed by electron energy loss spectroscopy (EELS) mapping with atomic resolution captured on a spherical aberration (*C_s_*)-corrected high-resolution scanning transmission electron microscope (HR−STEM) (Figure 7c). We also observed interesting core–shell phase replication behavior in this system. At the tip of the nanobranch, Pt atoms were observed in an *abcabc* stacking sequence, which is the packing structure of the *fcc* phase (Figure 7c,d). Therefore, on (0001) facets of the *hcp*−Ni, the Pt skin does not replicate the phase of the Ni substrate but takes its intrinsically stable *fcc* phase. On non-(0001) side facets of the Ni branch, the Pt atoms align in the same *abab* sequence as the Ni atoms in the core, suggesting successful phase replication. This is the first time that metastable *hcp*−Pt can be synthesized in a controllable manner. Therefore, this synthesis offers opportunities in the phase engineering of noble metal nanocrystals.

## 4. Applications of Unconventional Bimetallic Core–Shell Nanostructures

### 4.1. Enhancing the Stability of the Core Nanocrystals

**Figure 8:**
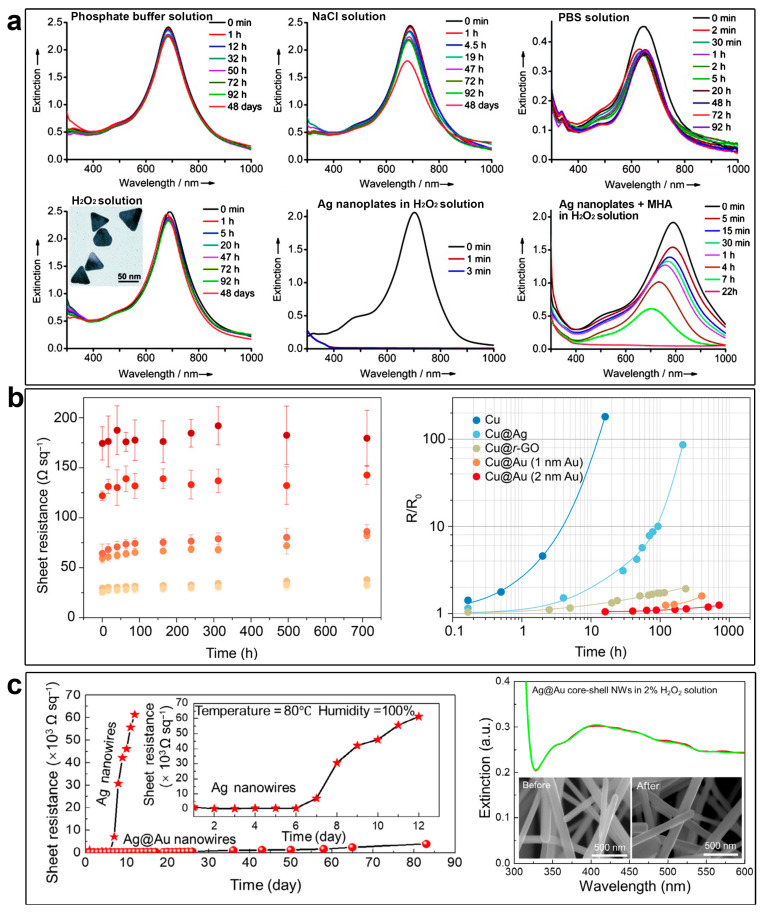
Bimetallic core–shell nanostructures with enhanced stability. (**a**) Chemical stability of Ag@Au core–shell nanoplates in different solutions. Reproduced from ref. [34]. Copyright (2012), with permission from Wiley-VCH. (**b**,**c**) Chemical and thermal stability of Cu@Au (**b**) and Ag@Au (**c**) core–shell nanowires. The star and spherical symbols in (**c**) indicate the resistances of films prepared with Ag and Ag@Au nanowires, respectively, changing with time. Reproduced from ref. [38]. Copyright (2017), with permission from the American Chemical Society. (**c**) Reproduced from ref. [62]. Copyright (2021), with permission from Springer Nature.

Due to their excellent optical and electrical properties, Ag and Cu nanostructures can be used in many fields, such as sensing and transparent conductors [59,60,61]. However, Ag and Cu are highly active metals and show weak chemical and thermal stability, which significantly limits their applications. Coating a thin layer of noble metal on the surface of Ag and Cu nanostructures is an effective way to improve their stability. Our group found that with the protection of a uniform Au layer, the Ag@Au nanoplates show excellent chemical stability against chemical etching (Figure 8a) [34]. As monitored by UV–vis spectra, the SPR bands of Ag@Au nanoplates were highly stable in a phosphate buffer solution, a NaCl solution, and a phosphate-buffered saline (PBS) solution, without showing a significant change in the position or intensity of the in-plane dipole SPR bands for four days, and even longer. By contrast, the pristine Ag nanoplates were quickly etched by PBS, NaCl, or H_2_O_2_, as evidenced by a significant shift in the peak position and a dramatic decrease in the intensity over a relatively short period. Yang and co-workers found that the stability of Cu nanowires can be greatly improved by coating a thin layer of Au (Figure 8b) [38]. The Cu@Au core–shell nanowires were fabricated on a flexible polyethylene terephthalate (PET). In 712 h of testing, the films maintained almost the same level of conductivity under harsh conditions (80 °C, 80 ± 5% humidity in the air). By contrast, the sheet resistance of pristine Cu nanowire mesh electrode showed a sharp increase in 3 h. A Au layer can also be used to prevent the oxidation of Ag nanowires. Yan and co-workers found that a Ag@Au core–shell nanowire mesh electrode exhibits significant enhancement in stability under heat (80 °C) and moisture (100% humidity) (Figure 8c) [62]. No significant change in sheet resistance was found for as long as 84 days. The UV–vis spectra further prove that Ag@Au core–shell nanowires show high stability during treatment with an H_2_O_2_ solution overnight.

### 4.2. Tuning the Optical Properties

**Figure 9:**
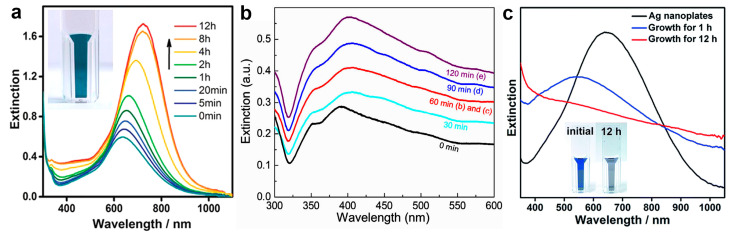
Bimetallic core–shell nanostructures with tunable optical properties. (**a**–**c**) Evolution of the UV–vis spectrum during the synthesis of the Ag@Au core–shell nanoplates (**a**); Ag@Au core–shell nanowires (**b**); and Ag@Pt core–shell nanoplates (**c**). (**a**) Reproduced from ref. [35]. Copyright (2015), with permission from Wiley-VCH. (**b**) Reproduced from ref. [62]. Copyright (2021), with permission from Springer Nature. (**c**) Reproduced from ref. [37]. Copyright (2018), with permission from the Royal Society of Chemistry.

The optical properties of Ag and Cu are closely related to their size, morphology, and surface properties [3,63,64,65,66], which provides the possibility for the regulation of their optical properties by coating a noble metal layer and building core–shell nanostructures. Our group and Yan’s group found that the localized surface plasmon resonance (LSPR) of Ag@Au core–shell nanocrystals had a red shift and an increasing intensity compared to Ag nanocrystals (Figure 9a,b) [35,62]. The red shift of the LSPR band can be attributed to the increase in the aspect ratio of the Ag@Au core–shell nanocrystals during epitaxial growth, and the increasing intensity can be attributed to the effective separation of the LSPR band from the interband transitions and the expanding volume of the nanoparticles. Conversely, the LSPR will decrease and blue shift when coated with a layer of Pt [34]. As shown in Figure 9c, the pristine Ag nanoplates showed an initial in-plane dipole-mode localized LSPR band at 640 nm of the wavelength. At the early stage of the epitaxial growth of Pt on the Ag nanoplates (after 1 h of growth), a blue shift of the LSPR can be witnessed with a decreased extinction efficiency. It is worth noting that galvanic replacement usually causes a red shift of the LSPR band due to the hollowing of the nanostructures. This distinct shift of the LSPR band confirmed the absence of galvanic replacement in the epitaxial growth. After the deposition of an appropriately thick layer of Pt (~2 nm, after 12 h of growth), the LSPR band disappeared in the visible range of the spectrum. As a result, the resulting Ag@Pt core–shell nanoplates displayed a grey/black color (Figure 9c). Therefore, the optical properties of the metal nanoplates can be utilized as an indicator to inspect the crystal growth process.

### 4.3. Improving the Catalytic Activities

**Figure 10:**
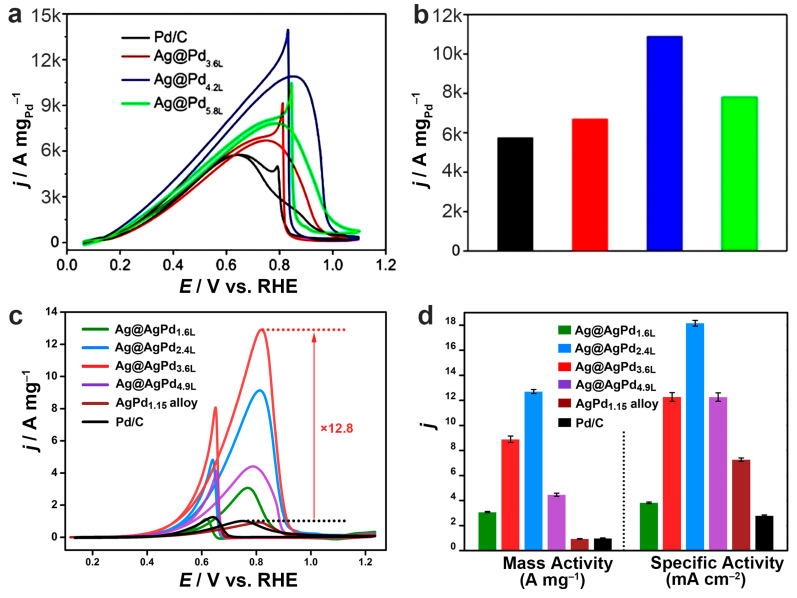
Bimetallic Ag@M core–shell nanostructures with improved catalytic activities. (**a**) FAOR performance of the Ag@Pd icosahedral nanocrystals. Electrolyte: N_2_-saturated 0.5 M HCOOH + 0.1 M HClO_4_ at 20 °C. (**b**) A comparison of the mass activities at anodic peak potential. Reproduced from ref. [46]. Copyright (2020), with permission from Wiley-VCH. (**c**) EOR performance of the Ag@AgPd core–shell nanoplates. Electrolyte: N_2_-saturated 1 M KOH + 1 M EtOH. (**d**) Specific and mass activities of the catalysts in terms of the anodic peak currents normalized to the mass of Pd and the ECSA. Reproduced from ref. [22]. Copyright (2021), with permission from the Royal Society of Chemistry.

Building a core–shell structure is a promising way to realize the regulation of the phase and electronic structure of noble metal catalysts [4,23,67]. The phase and facets of the catalysts can be easily tuned by the core–shell structure [21,25,68]. The difference in work function and electronegativity also makes the electronic properties easy to improve [17,21]. Moreover, the difference in atomic radius and lattice parameters between cores and shells makes it possible to build tensile or compressive strain in the shell [21,22,46]. Based on the above regulation, the optimization of the catalytic properties of noble metal nanomaterials can be realized. It is found that by using Ag as the core, tensile strain can be constructed in the noble metal shells for improved catalytic activities. For example, Ag@Pd core–shell icosahedral nanocrystals developed by Xia and co-workers exhibited enhanced activities in the electrocatalytic formic acid oxidation reaction (FAOR), which was explained by the electronic interactions between the Ag core and the Pd shell (Figure 10a) [46]. Our group disclosed the unambiguous efficacy of strong tensile strains in Pd, caused by Ag cores in a Ag@AgPd core–shell nanostructure, in improving both the reaction kinetics and the selectivity toward CO_2_ in the electrocatalytic ethanol oxidation reaction (EOR) (Figure 10b) [22]. The Pd atoms in the shell are found to take precisely the same arrangement as the Ag atoms in the core, leading to a substantial expansion of the lattice size by 4.5%. The tensile strain significantly strengthens the interaction between the catalyst and the intermediates involved in the EOR, leading to a substantial decrease in the energy barrier associated with the dehydration of ethanol of 39% and improved selectivity toward CO_2_ by 4.5 times, relative to the unstained monometallic Pd/C. As a result, the Ag@AgPd core–shell catalysts demonstrated a mass activity of 12.7 A mg_Pd_^−1^, 12.8 times greater than that of commercial Pd/C, making it an excellent catalyst for the EOR.

**Figure 11:**
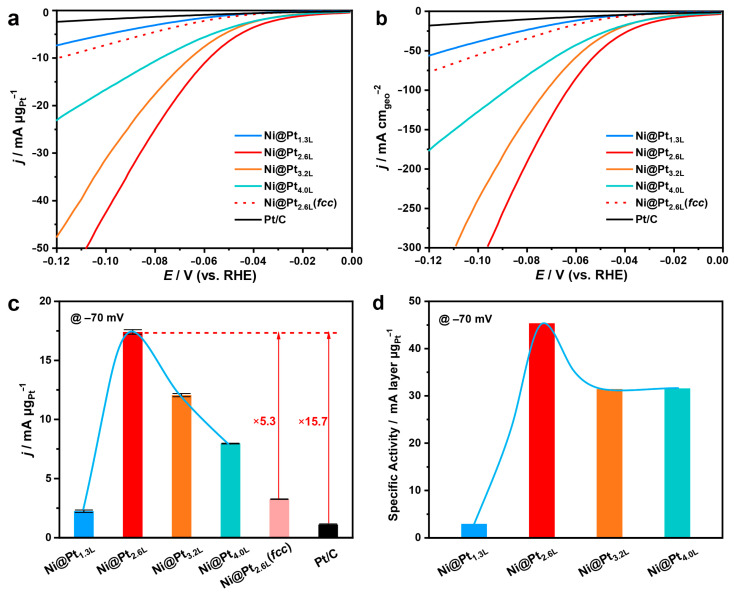
Bimetallic *hcp*−Ni@Pt core–shell nanostructures with improved catalytic activities. (**a**,**b**) LSV curves of the catalysts with 90% iR compensation in N_2_-saturated 1 M KOH at a scan rate of 10 mV s^−1^. (**c**,**d**) Mass and specific activities at −70 mV. Reproduced from ref. [21]. Copyright (2023), with permission from Springer Nature.

In addition, building 3d transition metal@noble metal core–shell nanostructures offers new opportunities in the phase engineering of noble metals for intriguing catalytic properties. Our group developed a synthesis for a metastable *hcp*−Pt phase by designing an *hcp*−Ni@Pt core–shell nanostructure and revealed its improved activities in the alkaline hydrogen evolution reaction (HER) (Figure 11) [21]. The metastable phase was proven critical in achieving excellent activities. The well-defined core–shell nanostructure further allows layer-by-layer electron transfer from the Ni core to the Pt skin, leading to modulated electronic properties. The experimental and DFT calculation results confirm that the energy barriers associated with the water dissociation and the hydrogen desorption processes are significantly reduced. With these features, the as-designed catalyst (approximately two atomic layers of *hcp*−Pt on Ni) demonstrated a high activity of 133 mA cm_geo_^−2^ and 17.4 mA μg_Pt_^−1^ at −70 mV in 1 M KOH.

### 4.4. Building Hollow Nanostructures or Open Frameworks

**Figure 12:**
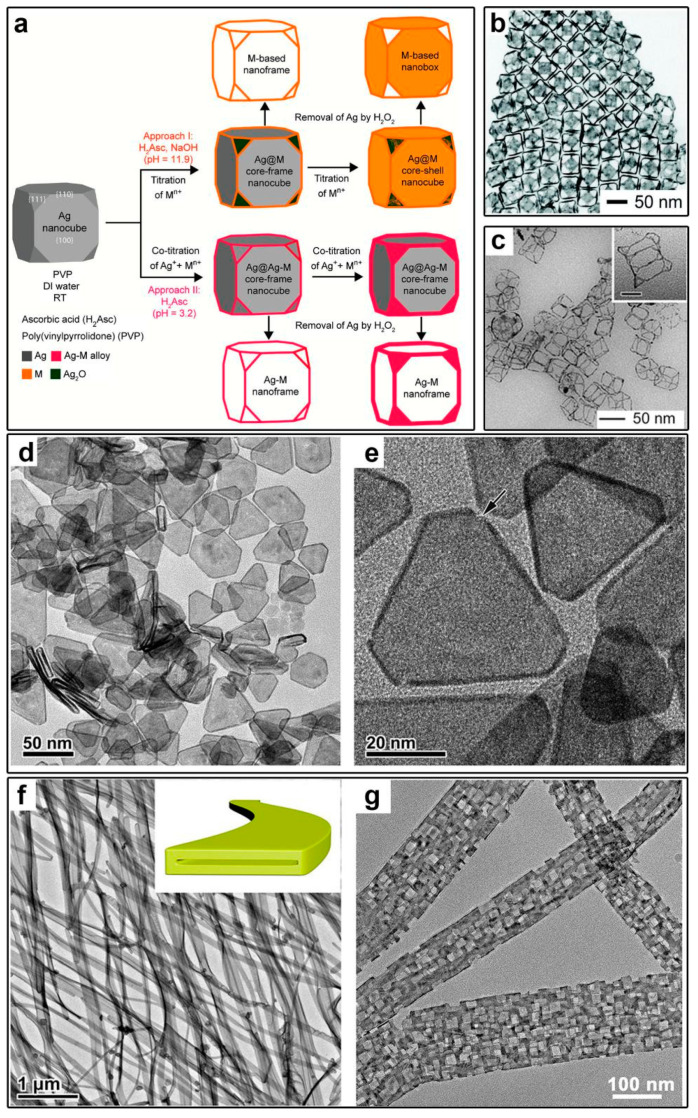
Synthesis of hollow nanostructures or open frameworks by etching the less-stable-metal cores from the core–shell nanostructure. (**a**) Two pathways for depositing a second metal M on a Ag nanocube in Ag@M core–frame and core–shell nanostructures, and eventual nanoframes and nanocages. Reproduced from ref. [44]. Copyright (2017), with permission from the American Chemical Society. (**b**) TEM image of Ag−Au{100} cuboctahedral nanoboxes. Reproduced from ref. [52]. Copyright (2020), with permission from the Royal Society of Chemistry. (**c**) TEM image of Ag−Pd nanoframes. Reproduced from ref. [51]. Copyright (2015), with permission from the American Chemical Society. (**d**,**e**) TEM images of ultrathin hollow Pt nanoplates. Reproduced from ref. [37]. Copyright (2018), with permission from the Royal Society of Chemistry. (**f**,**g**) TEM images of Pt−Ag nanotubes and porous nanoribbons after a ripening process. Reproduced from ref. [36]. Copyright (2018), with permission from the American Chemical Society.

Bimetallic core–shell nanostructures obtained from a galvanic-replacement-free synthesis can serve as starting materials for hollow nanostructures or open frameworks. Qin and co-workers synthesized such materials, including Au, Pd, and Pt nanocages, nanoboxes, and nanoframes, by etching the corresponding Ag@M bimetallic core–shell nanostructures (Figure 12a) [47,50,51,69]. For example, when M atoms were selectively deposited on the edges of a Ag nanocrystal, a Ag@M core–frame nanocrystal was formed. Nanoframes can be obtained by further etching of the Ag cores. Alternatively, when M atoms were conformally deposited on the entire Ag surface, nanoboxes or cages were obtained after removing the Ag cores (Figure 12b,c). Our group successfully synthesized hollow Pt nanoplates and nanotubes by etching Ag cores from Ag@Pt nanoplates and nanowires, respectively (Figure 12d–g) [36,37]. The morphology of the hollow nanostructures can be customized by choosing Ag nanocrystals of different shapes. The resulting nanostructures are composed of a Ag−Pt alloy, as confirmed by EDS, which can be caused by bimetallic intermixing during the synthesis and the etching processes. Interestingly, through a further thermal ripening process, porous hollow nanostructures can be obtained (Figure 12g). In particular, the nanopores in AgPd nanoribbons (or nanotubes) exhibit a regular rectangular shape, showing a typical edge length of ~8–10 nm. These unique structural and compositional features enabled excellent activity in the electrocatalytic methanol oxidation reaction (MOR).

## 5. Conclusions and Outlook

This review article briefly discussed the background and recent processes in preventing galvanic replacement reactions toward the synthesis of unconventional bimetallic core–shell nanostructures, each containing a less-stable-metal core and a noble metal shell. The applications of these unconventional core–shell nanostructures were also discussed. Overall, this unique technique will have a great impact on the synthetic chemistry of novel noble-metal-based nanostructures. Although great progress has been made in this direction, there remain great opportunities in designing new materials for SPR-based applications, nanodevices, and catalysis.

To date, Ag nanocrystals are the most common templates for the synthesis of unconventional core–shell nanostructures. Although Ag is cheaper than other noble metals, its price is still much higher than that of non-noble base metals. Creating bimetallic core–shell nanostructures with base metal cores is a promising route to creating noble metal-based materials with low costs and unique electronic properties. However, the synthesis is still in its infancy, with only very limited success till now. Challenges in the synthesis of such nanostructures are still to be addressed. In addition, the technique of preventing the galvanic replacement reaction is essential for creating multiple-shell nanostructures among many other exciting nanostructures. The different combinations and sequences of the metal shells may lead to unique optical and catalytic properties. Overall, we expect significant breakthroughs in these directions in the very near future.

## Figures and Tables

**Table 1:** Standard reduction potentials of representative metal salts at 25 °C [53].

Half Reaction	*E*^0^ (V)
Au^+^ + e^−^ = Au	1.83
Au^3+^ + 3e^−^ = Au	1.52
Pt^2+^ + 2e^−^ = Pt	1.188
Pd^2+^ + 2e^−^ = Pd	0.915
Ag^+^ + e^−^ = Ag	0.80
Cu^2+^ + 2e^−^ = Cu	0.34
Ni^2+^ + 2e^−^ = Ni	−0.257

**Table 2:** Standard reduction potentials of typical noble metal salts with different ligands at 25 °C [53].

Half Reaction	*E*^0^ (V)
Au^3+^ + 3e^−^ = Au	1.52
AuCl_4_^−^ + 3e^−^ = Au + 4Cl^−^	1.002
AuBr_4_^−^ + 3e^−^ = Au + 4Br^−^	0.854
AuI_4_^−^ + 3e^−^ = Au + 4I^−^	0.56
Au(SO_3_)_2_^3−^ + e^−^ = Au + 2SO_3_^2−^	0.111
Pt^2+^ + 2e^−^ = Pt	1.188
PtCl_4_^2−^ + 2e^−^ = Pt + 4Cl^−^	0.758
PtBr_4_^2−^ + 2e^−^ = Pt + 4Br^−^	0.698
Pd^2+^ + 2e^−^ = Pd	0.915
PdCl_4_^2−^ + 2e^−^ = Pd + 4Cl^−^	0.62
PdBr_4_^2−^ + 2e^−^ = Pd + 4Br^−^	0.49
Pd(NH_3_)_4_^2+^ + 2e^−^ = Pd + 4NH_3_	0.0

## Data Availability

Data sharing not applicable.
